# Benzyl 2-benzyl-4-[(3a*S*,7a*R*)-2,3,3a,4,5,6,7,7a-octa­hydro-1*H*-isoindol-2-yl]-4-oxobutano­ate

**DOI:** 10.1107/S1600536812037567

**Published:** 2012-09-05

**Authors:** Zhenhua Shang, Longpeng Xu, Ligang Zheng, Yong Zhang

**Affiliations:** a College of Chemical and Pharmaceutical Engineering, Hebei University of Science and Technology, Hebei Research Center of Pharmaceutical and Chemical Engineering, State Key Laboratory Breeding Base–Hebei Province Key Laboratory of Molecular Chemistry for Drugs, Shijiazhuang 050018, People’s Republic of China; b College of Chemical and Pharmaceutical Engineering, Hebei University of Science and Technology, Shijiazhuang 050018, People’s Republic of China; c Zhongqi Pharmacy (Shijiazhang), Shijiazhuang Pharmaceutical Group Co., Ltd (CSPC) , Shijiazhuang 050051, People’s Republic of China

## Abstract

In the title compound, C_26_H_31_NO_3_, the octa­hydro-1*H*-isoindole ring is not planar and the two rings are twisted with a C—C—C—C torsion angle of 73.6 (4)°. The six-membered ring has a chair conformation while the five-membered ring has an envelope conformation on the C-atom in position 7a. The H atoms in the 3a- and 7a-psitions are *cis* and the H—C—C—H torsion angle is 42.36°.

## Related literature

The title compound is an inter­mediate of mitiglinide, which was obtained when 2-benzyl-4-[(3a*S*,7a*R*)-2,3,3a,4,5,6,7,7a-octa­hydro-1*H*-isoindol-2-yl]-4-oxobutanoic acid was reacted with 1-(chloro­meth­yl)benzene in ethyl acetate with potassium iodide as catalyst. For the use of mitiglinide, a potassium channel antagonist, in the treatment of type 2 diabetes, see: Reimann *et al.* (2001 ▶).
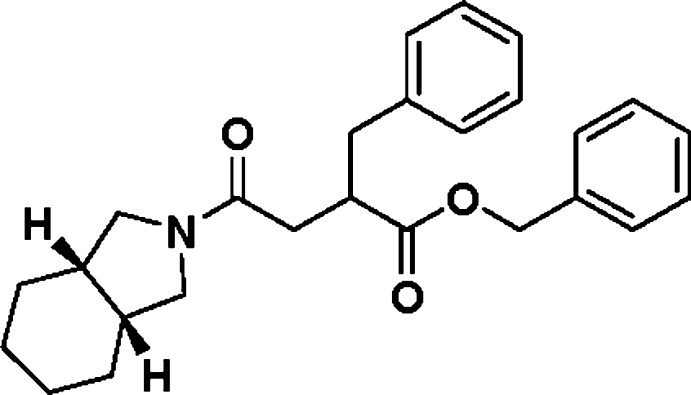


## Experimental

### 

#### Crystal data

C_26_H_31_NO_3_
*M* *_r_* = 405.52
Triclinic, 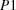

*a* = 5.8542 (12) Å
*b* = 9.0365 (18) Å
*c* = 11.352 (2) Å
α = 108.59 (3)°
β = 93.94 (3)°
γ = 101.23 (3)°
*V* = 552.7 (2) Å^3^
*Z* = 1
Mo *K*α radiation
μ = 0.08 mm^−1^
*T* = 294 K
0.16 × 0.14 × 0.10 mm

#### Data collection

Rigaku Saturn70 CCD diffractometer
Absorption correction: multi-scan (*CrystalClear*; Rigaku, 2005 ▶) *T*_min_ = 0.988, *T*_max_ = 0.992
5536 measured reflections
2588 independent reflections
1424 reflections with *I* > 2σ(*I*)
*R*_int_ = 0.049

#### Refinement

*R*[*F* ^2^ > 2σ(*F* ^2^)] = 0.043
*wR*(*F* ^2^) = 0.106
*S* = 0.89
2588 reflections
271 parameters
3 restraints
H-atom parameters constrained
Δρ_max_ = 0.18 e Å^−3^
Δρ_min_ = −0.20 e Å^−3^

### 

Data collection: *CrystalClear* (Rigaku, 2005 ▶); cell refinement: *CrystalClear*; data reduction: *CrystalClear*; program(s) used to solve structure: *SHELXS97* (Sheldrick, 2008 ▶); program(s) used to refine structure: *SHELXL97* (Sheldrick, 2008 ▶); molecular graphics: *SHELXTL* (Sheldrick, 2008 ▶); software used to prepare material for publication: *SHELXTL*.

## Supplementary Material

Crystal structure: contains datablock(s) global, I. DOI: 10.1107/S1600536812037567/hg5245sup1.cif

Structure factors: contains datablock(s) I. DOI: 10.1107/S1600536812037567/hg5245Isup2.hkl

Supplementary material file. DOI: 10.1107/S1600536812037567/hg5245Isup3.cml

Additional supplementary materials:  crystallographic information; 3D view; checkCIF report

## Figures and Tables

**Fig. 1.**
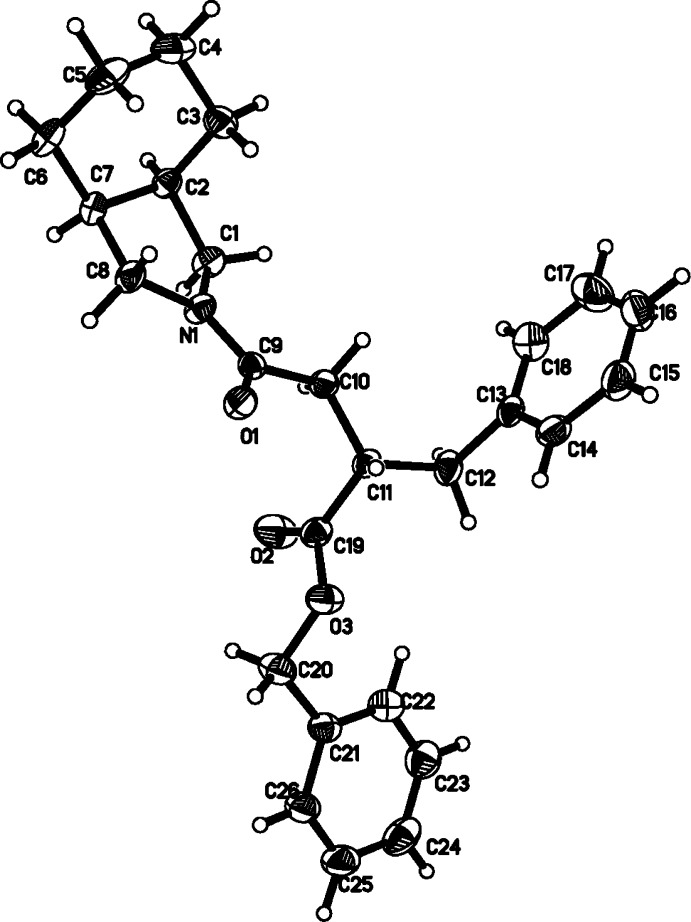
The molecular structure of the title compound, drawn with 30% probability ellipsoids.

## supplementary crystallographic information

### Comment

Mitiglinide, a potassium channel antagonist produced by Kissei Pharmaceutical Co., Ltd, was used for the treatment of type 2 diabetes mellitus (Reimann *et al.*, 2001). The title compound, as the key intermediate, was obtained When 2-benzyl-4-((3aS,7aR)-hexahydro -1*H*-isoindol-2(3*H*)-yl)-4-oxobutanoic acid was reacted with 1-(chloromethyl)benzene in ethyl acetate with potassium iodide as the catalyst. The octahydro-1*H*-isoindole ring is not on a plane and the two rings are twisted (C5—C6—C7—C8 = 73.6 (4)°, C3—C2—C1 = 112.5 (3)°, C4—C5—C6 = 111.2 (3)°). The 8-aza bicyclo[4.3.0]nonane ring is almost chair conformation (N1—C1—C2 = 102.8 (2)°, N1—C8—C7 = 105.1 (2)°), The C2 and C7 H atoms are *cis* form with the H2—C2—C7—H7 torsion angle 42.36°.

### Experimental

To a solution of 2-benzyl-4-((3aS,7aR)-hexahydro-1*H*-isoindol-2(3*H*)-yl) -4-oxobutanoic acid (13.8 g), KI (0.8 g) and potassium carbonate (6.6 g) in ethyl acetate (50 ml), 1-(chloromethyl)benzene (3.7 g) was added droply. After addition, the mixture was heated and reflux for 21 h. The reaction mixture was poured into 60 ml ice-water. The organic layer was seperated and distilled. When the ethyl acetate was recovered, the residue was added 120 ml petroleum ether. The product was obtained through filtration and recystalled. 50 mg of the title compound was dissolved in 50 ml ethanol and the solution was kept at room temperature for 10 d for natural evaporation. Colorless single crystals of the title compound obtained was suitable for X-ray analysis.

### Refinement

All H atoms attached to C atoms were fixed geometrically and treated as riding with C—H = 0.93 Å (aromatic), 0.98 Å (tertiary methyl CH group) or 0.97 Å (secondary CH_2_ group), with *U*_iso_(H)=1.2Ueq(C). The merging of the Friedel pairs are 3840–2588 = 1252.

### Crystal data

**Table:**

C_26_H_31_NO_3_	*Z* = 1
*M**_r_* = 405.52	*F*(000) = 218
Triclinic, *P*1	*D*_x_ = 1.218 Mg m^−^^3^
Hall symbol: P 1	Mo *K*α radiation, λ = 0.71073 Å
*a* = 5.8542 (12) Å	Cell parameters from 1681 reflections
*b* = 9.0365 (18) Å	θ = 1.9–27.9°
*c* = 11.352 (2) Å	µ = 0.08 mm^−^^1^
α = 108.59 (3)°	*T* = 294 K
β = 93.94 (3)°	Plate, colorless
γ = 101.23 (3)°	0.16 × 0.14 × 0.10 mm
*V* = 552.7 (2) Å^3^	

### Data collection

**Table:**

Rigaku Saturn70 CCD diffractometer	2588 independent reflections
Radiation source: rotating anode	1424 reflections with *I* > 2σ(*I*)
Multilayer monochromator	*R*_int_ = 0.049
Detector resolution: 7.31 pixels mm^-1^	θ_max_ = 27.9°, θ_min_ = 1.9°
ω scans	*h* = −7→7
Absorption correction: multi-scan (*CrystalClear*; Rigaku, 2005)	*k* = −11→10
*T*_min_ = 0.988, *T*_max_ = 0.992	*l* = −14→14
5536 measured reflections	

### Refinement

**Table:**

Refinement on *F*^2^	Primary atom site location: structure-invariant direct methods
Least-squares matrix: full	Secondary atom site location: difference Fourier map
*R*[*F*^2^ > 2σ(*F*^2^)] = 0.043	Hydrogen site location: inferred from neighbouring sites
*wR*(*F*^2^) = 0.106	H-atom parameters constrained
*S* = 0.89	*w* = 1/[σ^2^(*F*_o_^2^) + (0.0499*P*)^2^] where *P* = (*F*_o_^2^ + 2*F*_c_^2^)/3
2588 reflections	(Δ/σ)_max_ = 0.002
271 parameters	Δρ_max_ = 0.18 e Å^−^^3^
3 restraints	Δρ_min_ = −0.20 e Å^−^^3^

### Special details

**Table:**

Geometry. All e.s.d.'s (except the e.s.d. in the dihedral angle between two l.s. planes) are estimated using the full covariance matrix. The cell e.s.d.'s are taken into account individually in the estimation of e.s.d.'s in distances, angles and torsion angles; correlations between e.s.d.'s in cell parameters are only used when they are defined by crystal symmetry. An approximate (isotropic) treatment of cell e.s.d.'s is used for estimating e.s.d.'s involving l.s. planes.
Refinement. Refinement of *F*^2^ against ALL reflections. The weighted *R*-factor *wR* and goodness of fit *S* are based on *F*^2^, conventional *R*-factors *R* are based on *F*, with *F* set to zero for negative *F*^2^. The threshold expression of *F*^2^ > σ(*F*^2^) is used only for calculating *R*-factors(gt) *etc*. and is not relevant to the choice of reflections for refinement. *R*-factors based on *F*^2^ are statistically about twice as large as those based on *F*, and *R*- factors based on ALL data will be even larger.

### Fractional atomic coordinates and isotropic or equivalent isotropic displacement parameters (Å^2^)

**Table:**

	*x*	*y*	*z*	*U*_iso_*/*U*_eq_	
O1	−0.1006 (3)	0.2391 (3)	0.4493 (2)	0.0461 (6)	
O2	0.2379 (5)	0.2088 (3)	0.7354 (3)	0.0781 (9)	
O3	0.0275 (4)	0.3929 (3)	0.7968 (2)	0.0530 (6)	
N1	0.1082 (4)	0.0726 (3)	0.3389 (2)	0.0414 (7)	
C1	0.3247 (5)	0.0184 (4)	0.3070 (3)	0.0460 (8)	
H1A	0.3538	−0.0562	0.3485	0.055*	
H1B	0.4599	0.1083	0.3300	0.055*	
C2	0.2709 (5)	−0.0637 (4)	0.1651 (3)	0.0388 (7)	
H2	0.3633	−0.1449	0.1378	0.047*	
C3	0.3173 (6)	0.0541 (4)	0.0949 (3)	0.0500 (9)	
H3A	0.2529	0.1458	0.1348	0.060*	
H3B	0.4858	0.0921	0.1003	0.060*	
C4	0.2112 (7)	−0.0190 (5)	−0.0407 (4)	0.0660 (11)	
H4A	0.2796	−0.1082	−0.0821	0.079*	
H4B	0.2458	0.0602	−0.0819	0.079*	
C5	−0.0515 (7)	−0.0771 (5)	−0.0518 (4)	0.0683 (12)	
H5A	−0.1179	−0.1232	−0.1398	0.082*	
H5B	−0.1199	0.0131	−0.0131	0.082*	
C6	−0.1126 (6)	−0.2014 (4)	0.0109 (3)	0.0551 (10)	
H6A	−0.0674	−0.2984	−0.0369	0.066*	
H6B	−0.2816	−0.2274	0.0102	0.066*	
C7	0.0077 (5)	−0.1453 (4)	0.1451 (3)	0.0422 (8)	
H7	−0.0069	−0.2393	0.1719	0.051*	
C8	−0.0910 (5)	−0.0199 (4)	0.2404 (3)	0.0452 (8)	
H8A	−0.1499	0.0490	0.2014	0.054*	
H8B	−0.2179	−0.0713	0.2748	0.054*	
C9	0.0902 (5)	0.2018 (4)	0.4337 (3)	0.0352 (7)	
C10	0.3132 (5)	0.3000 (4)	0.5201 (3)	0.0392 (7)	
H10A	0.4084	0.3610	0.4772	0.047*	
H10B	0.4018	0.2282	0.5389	0.047*	
C11	0.2690 (5)	0.4157 (4)	0.6433 (3)	0.0383 (7)	
H11	0.1502	0.4708	0.6243	0.046*	
C12	0.4942 (6)	0.5424 (4)	0.7134 (3)	0.0505 (9)	
H12A	0.4676	0.5993	0.7976	0.061*	
H12B	0.6203	0.4888	0.7205	0.061*	
C13	0.5688 (5)	0.6615 (4)	0.6478 (3)	0.0400 (7)	
C14	0.4546 (6)	0.7839 (4)	0.6598 (3)	0.0517 (9)	
H14	0.3265	0.7878	0.7040	0.062*	
C15	0.5261 (7)	0.9007 (4)	0.6076 (3)	0.0639 (11)	
H15	0.4469	0.9826	0.6171	0.077*	
C16	0.7108 (7)	0.8964 (5)	0.5428 (4)	0.0687 (11)	
H16	0.7600	0.9756	0.5082	0.082*	
C17	0.8251 (7)	0.7751 (6)	0.5284 (4)	0.0755 (13)	
H17	0.9516	0.7715	0.4831	0.091*	
C18	0.7548 (6)	0.6577 (5)	0.5804 (4)	0.0589 (10)	
H18	0.8339	0.5756	0.5697	0.071*	
C19	0.1781 (6)	0.3255 (4)	0.7267 (3)	0.0439 (8)	
C20	−0.0779 (7)	0.3150 (5)	0.8789 (3)	0.0586 (10)	
H20A	−0.0677	0.2035	0.8495	0.070*	
H20B	−0.2432	0.3172	0.8749	0.070*	
C21	0.0366 (6)	0.3918 (4)	1.0121 (3)	0.0465 (8)	
C22	0.2699 (6)	0.4696 (5)	1.0456 (4)	0.0586 (10)	
H22	0.3625	0.4787	0.9838	0.070*	
C23	0.3670 (8)	0.5339 (5)	1.1696 (4)	0.0681 (11)	
H23	0.5252	0.5862	1.1909	0.082*	
C24	0.2340 (9)	0.5220 (5)	1.2627 (4)	0.0724 (12)	
H24	0.3005	0.5667	1.3467	0.087*	
C25	0.0015 (9)	0.4432 (5)	1.2297 (4)	0.0730 (13)	
H25	−0.0898	0.4315	1.2915	0.088*	
C26	−0.0973 (7)	0.3812 (4)	1.1053 (4)	0.0564 (9)	
H26	−0.2565	0.3316	1.0840	0.068*	

### Atomic displacement parameters (Å^2^)

**Table:**

	*U*^11^	*U*^22^	*U*^33^	*U*^12^	*U*^13^	*U*^23^
O1	0.0369 (12)	0.0497 (13)	0.0454 (14)	0.0100 (10)	0.0056 (10)	0.0075 (11)
O2	0.122 (2)	0.0620 (17)	0.083 (2)	0.0488 (17)	0.0506 (18)	0.0457 (16)
O3	0.0657 (15)	0.0563 (15)	0.0471 (15)	0.0236 (12)	0.0191 (12)	0.0233 (12)
N1	0.0273 (13)	0.0517 (16)	0.0349 (15)	0.0075 (12)	−0.0012 (11)	0.0028 (13)
C1	0.0328 (17)	0.056 (2)	0.044 (2)	0.0137 (14)	0.0036 (15)	0.0085 (16)
C2	0.0368 (16)	0.0396 (18)	0.0369 (18)	0.0121 (13)	0.0034 (13)	0.0070 (14)
C3	0.0440 (18)	0.055 (2)	0.055 (2)	0.0111 (16)	0.0147 (16)	0.0213 (18)
C4	0.088 (3)	0.079 (3)	0.042 (2)	0.028 (2)	0.017 (2)	0.028 (2)
C5	0.084 (3)	0.079 (3)	0.035 (2)	0.025 (2)	−0.0119 (19)	0.010 (2)
C6	0.057 (2)	0.048 (2)	0.042 (2)	0.0109 (17)	−0.0080 (17)	−0.0052 (17)
C7	0.0438 (18)	0.0321 (17)	0.042 (2)	0.0040 (14)	0.0001 (15)	0.0054 (15)
C8	0.0309 (16)	0.052 (2)	0.0377 (19)	0.0009 (14)	−0.0017 (14)	0.0019 (15)
C9	0.0310 (15)	0.0403 (17)	0.0323 (17)	0.0027 (13)	0.0034 (12)	0.0130 (14)
C10	0.0348 (16)	0.0454 (18)	0.0341 (18)	0.0055 (13)	0.0028 (13)	0.0116 (15)
C11	0.0441 (17)	0.0372 (17)	0.0297 (17)	0.0082 (14)	−0.0007 (14)	0.0082 (14)
C12	0.062 (2)	0.0398 (19)	0.042 (2)	0.0000 (16)	−0.0092 (16)	0.0134 (16)
C13	0.0426 (18)	0.0352 (17)	0.0335 (17)	0.0024 (13)	−0.0067 (14)	0.0063 (13)
C14	0.067 (2)	0.046 (2)	0.042 (2)	0.0200 (18)	0.0127 (17)	0.0092 (16)
C15	0.087 (3)	0.041 (2)	0.062 (3)	0.018 (2)	0.007 (2)	0.015 (2)
C16	0.079 (3)	0.055 (2)	0.068 (3)	−0.003 (2)	0.002 (2)	0.028 (2)
C17	0.048 (2)	0.097 (4)	0.082 (3)	0.005 (2)	0.017 (2)	0.038 (3)
C18	0.049 (2)	0.059 (2)	0.070 (3)	0.0163 (18)	0.0054 (19)	0.022 (2)
C19	0.0541 (19)	0.0417 (19)	0.0372 (19)	0.0149 (16)	0.0051 (15)	0.0132 (15)
C20	0.059 (2)	0.072 (3)	0.052 (2)	0.0118 (19)	0.0156 (19)	0.030 (2)
C21	0.058 (2)	0.044 (2)	0.047 (2)	0.0195 (17)	0.0160 (17)	0.0224 (17)
C22	0.061 (2)	0.065 (2)	0.053 (2)	0.018 (2)	0.0123 (19)	0.021 (2)
C23	0.074 (3)	0.062 (3)	0.067 (3)	0.017 (2)	0.005 (2)	0.021 (2)
C24	0.114 (4)	0.057 (3)	0.047 (3)	0.030 (3)	0.000 (3)	0.015 (2)
C25	0.115 (4)	0.059 (3)	0.057 (3)	0.026 (3)	0.038 (3)	0.028 (2)
C26	0.069 (2)	0.053 (2)	0.055 (2)	0.0170 (18)	0.023 (2)	0.0240 (19)

### Geometric parameters (Å, º)

**Table:**

O1—C9	1.236 (3)	C10—H10B	0.9700
O2—C19	1.204 (4)	C11—C19	1.495 (5)
O3—C19	1.334 (4)	C11—C12	1.539 (4)
O3—C20	1.440 (4)	C11—H11	0.9800
N1—C9	1.340 (4)	C12—C13	1.509 (5)
N1—C8	1.468 (4)	C12—H12A	0.9700
N1—C1	1.473 (4)	C12—H12B	0.9700
C1—C2	1.524 (4)	C13—C18	1.373 (5)
C1—H1A	0.9700	C13—C14	1.376 (4)
C1—H1B	0.9700	C14—C15	1.379 (5)
C2—C3	1.516 (4)	C14—H14	0.9300
C2—C7	1.541 (4)	C15—C16	1.348 (5)
C2—H2	0.9800	C15—H15	0.9300
C3—C4	1.501 (5)	C16—C17	1.364 (6)
C3—H3A	0.9700	C16—H16	0.9300
C3—H3B	0.9700	C17—C18	1.381 (6)
C4—C5	1.507 (6)	C17—H17	0.9300
C4—H4A	0.9700	C18—H18	0.9300
C4—H4B	0.9700	C20—C21	1.493 (5)
C5—C6	1.512 (5)	C20—H20A	0.9700
C5—H5A	0.9700	C20—H20B	0.9700
C5—H5B	0.9700	C21—C22	1.374 (5)
C6—C7	1.515 (5)	C21—C26	1.375 (4)
C6—H6A	0.9700	C22—C23	1.372 (5)
C6—H6B	0.9700	C22—H22	0.9300
C7—C8	1.532 (4)	C23—C24	1.374 (6)
C7—H7	0.9800	C23—H23	0.9300
C8—H8A	0.9700	C24—C25	1.372 (7)
C8—H8B	0.9700	C24—H24	0.9300
C9—C10	1.507 (4)	C25—C26	1.379 (6)
C10—C11	1.529 (4)	C25—H25	0.9300
C10—H10A	0.9700	C26—H26	0.9300
			
C19—O3—C20	117.8 (3)	C11—C10—H10B	108.9
C9—N1—C8	121.5 (2)	H10A—C10—H10B	107.7
C9—N1—C1	127.0 (2)	C19—C11—C10	110.2 (3)
C8—N1—C1	110.9 (2)	C19—C11—C12	109.0 (3)
N1—C1—C2	102.8 (2)	C10—C11—C12	111.7 (3)
N1—C1—H1A	111.2	C19—C11—H11	108.6
C2—C1—H1A	111.2	C10—C11—H11	108.6
N1—C1—H1B	111.2	C12—C11—H11	108.6
C2—C1—H1B	111.2	C13—C12—C11	112.4 (3)
H1A—C1—H1B	109.1	C13—C12—H12A	109.1
C3—C2—C1	112.5 (3)	C11—C12—H12A	109.1
C3—C2—C7	111.5 (3)	C13—C12—H12B	109.1
C1—C2—C7	102.8 (3)	C11—C12—H12B	109.1
C3—C2—H2	109.9	H12A—C12—H12B	107.9
C1—C2—H2	109.9	C18—C13—C14	117.9 (3)
C7—C2—H2	109.9	C18—C13—C12	122.6 (3)
C4—C3—C2	112.4 (3)	C14—C13—C12	119.5 (3)
C4—C3—H3A	109.1	C13—C14—C15	121.3 (3)
C2—C3—H3A	109.1	C13—C14—H14	119.3
C4—C3—H3B	109.1	C15—C14—H14	119.3
C2—C3—H3B	109.1	C16—C15—C14	120.1 (3)
H3A—C3—H3B	107.8	C16—C15—H15	119.9
C3—C4—C5	110.2 (3)	C14—C15—H15	119.9
C3—C4—H4A	109.6	C15—C16—C17	119.7 (4)
C5—C4—H4A	109.6	C15—C16—H16	120.2
C3—C4—H4B	109.6	C17—C16—H16	120.2
C5—C4—H4B	109.6	C16—C17—C18	120.6 (4)
H4A—C4—H4B	108.1	C16—C17—H17	119.7
C4—C5—C6	111.2 (3)	C18—C17—H17	119.7
C4—C5—H5A	109.4	C13—C18—C17	120.4 (3)
C6—C5—H5A	109.4	C13—C18—H18	119.8
C4—C5—H5B	109.4	C17—C18—H18	119.8
C6—C5—H5B	109.4	O2—C19—O3	122.4 (3)
H5A—C5—H5B	108.0	O2—C19—C11	125.2 (3)
C5—C6—C7	112.8 (3)	O3—C19—C11	112.3 (3)
C5—C6—H6A	109.0	O3—C20—C21	112.9 (3)
C7—C6—H6A	109.0	O3—C20—H20A	109.0
C5—C6—H6B	109.0	C21—C20—H20A	109.0
C7—C6—H6B	109.0	O3—C20—H20B	109.0
H6A—C6—H6B	107.8	C21—C20—H20B	109.0
C6—C7—C8	115.5 (3)	H20A—C20—H20B	107.8
C6—C7—C2	114.9 (3)	C22—C21—C26	118.7 (3)
C8—C7—C2	102.4 (2)	C22—C21—C20	123.1 (3)
C6—C7—H7	107.8	C26—C21—C20	118.2 (3)
C8—C7—H7	107.8	C23—C22—C21	120.5 (4)
C2—C7—H7	107.8	C23—C22—H22	119.8
N1—C8—C7	105.1 (2)	C21—C22—H22	119.8
N1—C8—H8A	110.7	C22—C23—C24	120.9 (4)
C7—C8—H8A	110.7	C22—C23—H23	119.5
N1—C8—H8B	110.7	C24—C23—H23	119.5
C7—C8—H8B	110.7	C25—C24—C23	118.9 (4)
H8A—C8—H8B	108.8	C25—C24—H24	120.6
O1—C9—N1	121.5 (3)	C23—C24—H24	120.6
O1—C9—C10	121.5 (3)	C24—C25—C26	120.2 (4)
N1—C9—C10	117.0 (2)	C24—C25—H25	119.9
C9—C10—C11	113.3 (2)	C26—C25—H25	119.9
C9—C10—H10A	108.9	C21—C26—C25	120.8 (4)
C11—C10—H10A	108.9	C21—C26—H26	119.6
C9—C10—H10B	108.9	C25—C26—H26	119.6
			
C9—N1—C1—C2	−149.7 (3)	C10—C11—C12—C13	70.1 (3)
C8—N1—C1—C2	21.3 (3)	C11—C12—C13—C18	−104.7 (4)
N1—C1—C2—C3	83.2 (3)	C11—C12—C13—C14	78.3 (4)
N1—C1—C2—C7	−36.9 (3)	C18—C13—C14—C15	−1.0 (5)
C1—C2—C3—C4	−166.1 (3)	C12—C13—C14—C15	176.1 (3)
C7—C2—C3—C4	−51.2 (4)	C13—C14—C15—C16	0.3 (5)
C2—C3—C4—C5	59.6 (4)	C14—C15—C16—C17	0.5 (6)
C3—C4—C5—C6	−59.5 (4)	C15—C16—C17—C18	−0.6 (6)
C4—C5—C6—C7	52.5 (4)	C14—C13—C18—C17	0.9 (5)
C5—C6—C7—C8	73.6 (4)	C12—C13—C18—C17	−176.1 (4)
C5—C6—C7—C2	−45.4 (4)	C16—C17—C18—C13	−0.2 (6)
C3—C2—C7—C6	44.2 (4)	C20—O3—C19—O2	−4.4 (5)
C1—C2—C7—C6	165.0 (3)	C20—O3—C19—C11	178.4 (3)
C3—C2—C7—C8	−81.8 (3)	C10—C11—C19—O2	36.0 (4)
C1—C2—C7—C8	38.9 (3)	C12—C11—C19—O2	−86.9 (4)
C9—N1—C8—C7	174.7 (3)	C10—C11—C19—O3	−146.9 (3)
C1—N1—C8—C7	3.2 (3)	C12—C11—C19—O3	90.2 (3)
C6—C7—C8—N1	−151.8 (3)	C19—O3—C20—C21	100.6 (4)
C2—C7—C8—N1	−26.1 (3)	O3—C20—C21—C22	−29.7 (5)
C8—N1—C9—O1	7.4 (4)	O3—C20—C21—C26	151.6 (3)
C1—N1—C9—O1	177.6 (3)	C26—C21—C22—C23	0.6 (5)
C8—N1—C9—C10	−172.3 (3)	C20—C21—C22—C23	−178.1 (3)
C1—N1—C9—C10	−2.2 (4)	C21—C22—C23—C24	0.0 (6)
O1—C9—C10—C11	15.8 (4)	C22—C23—C24—C25	0.6 (6)
N1—C9—C10—C11	−164.5 (3)	C23—C24—C25—C26	−1.8 (6)
C9—C10—C11—C19	73.2 (3)	C22—C21—C26—C25	−1.9 (5)
C9—C10—C11—C12	−165.5 (2)	C20—C21—C26—C25	176.9 (4)
C19—C11—C12—C13	−167.8 (3)	C24—C25—C26—C21	2.5 (6)
